# The Effect of Heat Waves on Mental Health in a Temperate Australian City

**DOI:** 10.1289/ehp.11339

**Published:** 2008-06-30

**Authors:** Alana Hansen, Peng Bi, Monika Nitschke, Philip Ryan, Dino Pisaniello, Graeme Tucker

**Affiliations:** 1 Discipline of Public Health, School of Population Health and Clinical Practice, Faculty of Health Sciences, The University of Adelaide, Adelaide, South Australia, Australia; 2 South Australian Department of Health, Adelaide, South Australia, Australia

**Keywords:** dementia, heat waves, mental health, psychiatric, schizophrenia, temperature, weather

## Abstract

**Objective:**

The goal of this study was to identify mental, behavioral, and cognitive disorders that may be triggered or exacerbated during heat waves, predisposing individuals to heat-related morbidity and mortality.

**Design:**

Using health outcome data from Adelaide, South Australia, for 1993–2006, we estimated the effect of heat waves on hospital admissions and mortalities attributed to mental, behavioral, and cognitive disorders. We analyzed data using Poisson regression accounting for overdispersion and controlling for season and long-term trend, and we performed threshold analysis using hockey stick regression.

**Results:**

Above a threshold of 26.7°C, we observed a positive association between ambient temperature and hospital admissions for mental and behavioral disorders. Compared with non–heat-wave periods, hospital admissions increased by 7.3% during heat waves. Specific illnesses for which admissions increased included organic illnesses, including symptomatic mental disorders; dementia; mood (affective) disorders; neurotic, stress related, and somatoform disorders; disorders of psychological development; and senility. Mortalities attributed to mental and behavioral disorders increased during heat waves in the 65- to 74-year age group and in persons with schizophrenia, schizotypal, and delusional disorders. Dementia deaths increased in those up to 65 years of age.

**Conclusion:**

Our results suggest that episodes of extreme heat pose a salient risk to the health and well-being of the mentally ill.

Relevance to Clinical or Professional Practice: Improvements in the management and care of the mentally ill need to be addressed to avoid an increase in psychiatric morbidity and mortality as heat waves become more frequent.

Compared with the general population, persons with mental health problems often experience poorer overall health with higher rates of morbidity and mortality (Australian Bureau of Statistics 2006). Additionally, it has been well documented that, because of behavioral issues and medications that interfere with physiological homeostasis, those with mental illness are susceptible to the effects of extreme heat, as demonstrated by increases in hospital admissions (Kovats and Ebi 2006; Shiloh et al. 2005) and mortalities (Bark 1998; Basu and Samet 2002; Kaiser et al. 2001; Naughton et al. 2002) associated with heat waves. However, few studies have characterized specific mental and behavioral disorders (MBDs) that may be exacerbated by high temperatures.

Mental illnesses may range from short-term bouts of depression and anxiety to long-term conditions such as developmental impairments, chronic depression, schizophrenia, or chronic anxiety disorders [Australian Institute of Health and Welfare (AIHW) 2006]. Accounting for an estimated 13% of the national disease burden in Australia, mental illness is among the 10 leading causes of disease in 2003 and has been declared a National Health Priority Area (AIHW 2006). In the State of South Australia, studies have shown that as many as one in five adults has a mental problem (Taylor et al. 1999).

Our aim in this study was to identify mental disorders that contribute to heat-related morbidity and mortality in a temperate climatic region. This study is unique in that to our knowledge, no similar investigation has previously been undertaken. Moreover, we used both hospital admissions and mortality data spanning a 13-year period to establish a comprehensive overview of the temperature–health association. With mental disorders causing an already significant burden on the public health system, understanding the relationship between hot-weather extremes and psychiatric illness will assist in identifying populations at risk as global warming ensues, and provide valuable information for decision makers in the mental health and social service sectors.

## Materials and Methods

The city of Adelaide, with a population of 1.16 million (Australian Bureau of Statistics 2008), is the capital of the State of South Australia and is situated near the coast in the temperate region of southern Australia (McMichael and Beers 1994). The city’s climatic conditions include mild winters and long, hot, dry summers during which heat waves are common. Temperatures exceeding 40°C occur on average 3 days/year (Government of South Australia 2008), making the climate ideal for the study of the health effects of heat waves.

We obtained morbidity data for the Adelaide metropolitan area for the period 1 July 1993 to 30 June 2006 from the South Australian Department of Health (Adelaide, South Australia, Australia). We accessed principal hospital discharge diagnoses using the Integrated South Australian Activity Collection (ISAAC), an official collection of admitted patient activity in the state’s public and private hospitals (Government of South Australia 2002). Data relating to individuals who resided outside of the Adelaide metropolitan area were excluded from the study. We obtained mortality data for the period 1 July 1993 to 22 December 2004 from the Australian Bureau of Statistics. Underlying causes of death were coded by the Australian Bureau of Statistics using computer-assisted coding.

Daily counts of admissions and mortalities with principal discharge diagnoses or cause of death, respectively, attributed to MBDs were accessed. MBDs were defined in accordance with the *International Classification of Diseases, 10th Revision* [ICD-10; World Health Organization (WHO) 2007]. Subclassifications of MBDs (ICD-10 codes F00–F99) included in the study are shown in Table 1. Dementia was identified separately, although it is already included within the classification of organic, including symptomatic, mental disorders. Additional to the aforementioned disorders, we included Alzheimer’s disease, senile degeneration of the brain, and senility to account for age-related cognitive disorders not captured within the F00–F99 subclassifications.

We obtained climatic data from the Australian Bureau of Meteorology, Adelaide (Kent Town). Daily maximum ambient air temperatures for Adelaide were accessed from a central city weather station considered to be representative of conditions across the total metropolitan area, as advised by the Bureau of Meteorology.

### Data analyses

We analyzed hospital admissions and mortality data collectively and with stratification by age and sex. Seasons were defined as cool (1 April to 30 September) and warm (1 October to 31 March). Because the focus of the study was heat-related morbidity and mortality, we used only the warm season in the analysis. We defined heat waves as ≥3 consecutive days when the daily maximum temperature (*T*_max_) reached or exceeded 35°C, as in similar studies (Nitschke et al. 2007).

We used conditional fixed-effects Poisson regression models to quantify the association with heat waves, with the referent period being all non–heat-wave days during the warm season. A goodness-of-fit test was applied to each model, and if significant overdispersion was detected, we used negative binomial maximum-likelihood regression (Glynn and Buring 1996). By performing within-year analyses, we adjusted for long-term trends (Nitschke et al. 2007). Results are expressed as incidence rate ratios (IRRs) with 95% confidence intervals (CIs).

We explored the relationship between daily admissions or mortalities and *T*_max_ across both seasons graphically using a lowess smoother, which performs locally weighted regressions of the *y* variable on the *x* variable to calculate smoothed data-point values (StataCorp 2005). We determined the existence of a threshold by inspecting the plots; if a threshold was present, it was estimated visually and confirmed quantitatively using a nonlinear least-squares estimation incorporating the nl function in Stata, version 9.2 (StataCorp 2005). Based on the assumption of more than one segment in the relationship between two variables, this “hockey stick” method (Chuang et al. 2000; Yanagimoto and Yamamoto 1979; Zhang et al. 2007) uses a function evaluator program to determine the cut point in the curve at which the change in slope occurs, providing this value is common to both functions. Parameters are determined using informative initial estimates of the slope and threshold values in iterative nonlinear regression models. Calculated threshold values should approximate visual estimates and have a statistical significance level of 0.05.

The threshold model can be defined as follows:


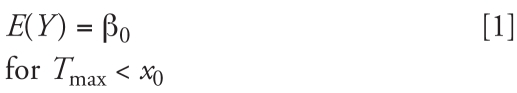



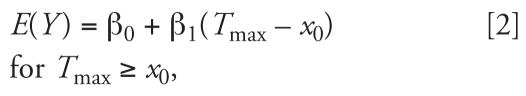


, where *E*(*Y*) is the expected value of the outcome variable, β_0_ is the baseline number of daily admissions, *x*_0_ is the threshold temperature value to be determined, β_1_ is the slope of the segment of the regression line prior to the changepoint; β_0_ is the slope after the change-point. If a nonzero slope is expected in Equation 1, the model becomes


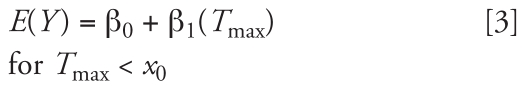



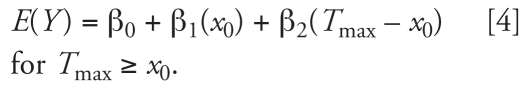


## Results

Hospital admission data spanned the period 1 July 1993 to 30 June 2006, and mortality data were obtained for 1 July 1993 to 22 December 2004. During this time, there were 31 and 29 heat waves, respectively, ranging in duration from 3 to 8 days, including an extreme heat event in January 2006 with temperatures over 4 continuous days exceeding 40°C. The highest recorded maximum temperature was 44.3°C during an 8-day heat wave in 2004.

### Hospital admissions

**Figure 1:**
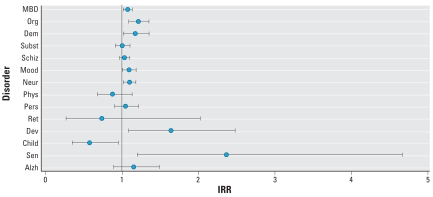
Calculated point estimates (IRRs and 95% CIs) of the risk during heat waves compared with non-heat-wave periods, of hospital admission for mental, behavioral, and cognitive disorders. Abbreviations: Alzh, Alzheimer’s disease; Child, behavioral and emotional disorders with onset usually occurring in childhood and adolescence; Dem, dementia; Dev, disorders of psychological development; Mood, mood (affective) disorders; Neur, neurotic, stress-related, and somatoform disorders; Org, organic, including symptomatic, mental disorders; Pers, disorders of adult personality and behavior; Phys, behavioral syndromes associated with physiological disturbances and physical factors; Ret, mental retardation; Schiz, schizophrenia, schizotypal, and delusional disorders; Sen, senility; Subst, disorders due to psychoactive substance use.

Regression results showed that 7 of the 15 categories of mental and cognitive disorders studied showed no significant association of hospital admissions with heat waves (Table 1, Figure 1); however, 2 of these 7 showed age- or sex-specific associations (Table 2). Therefore, 5 categories had no significant association of hospital admissions with heat waves: schizophrenia, schizotypal, and delusional disorders (ICD-10 codes F20–F29); disorders of adult personality and behavior (F60–F69); mental retardation (F70–F79); Alzheimer’s disease (G30–G30.9); and senile degeneration of the brain not elsewhere classified (G31.1). Behavioral and emotional disorders with onset usually occurring in childhood and adolescence (F90–F98) showed a statistically significant decrease in admissions during heat waves compared with non–heat-wave periods. Increases in hospital admissions for the remaining categories were found to occur during heat-wave periods, as discussed below.

#### MBDs (ICD-10 codes F00–F99)

**Figure 2:**
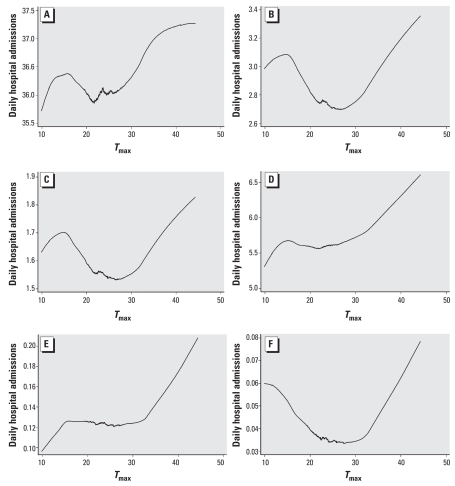
Exposure–response relationships between T_max_ and hospital admissions for mental disorders for (*A*) All MBDs; (*B*) organic, including symptomatic, mental disorders; (*C*) dementia; (*D*) neurotic, stress-related, and somatoform disorders; (*E*) disorders of psychological development; and (*F*) senility. Data were smoothed using a lowess smoother, bandwidth = 0.8.

During the study period, a total of 171,614 admissions to Adelaide hospitals occurred with the principal discharge diagnoses of MBDs (F00–F99). Of these, 4,629 admissions occurred during heat waves, representing a daily mean of 38.6 admissions compared with 35.8 during the warm season. We observed the exposure–response association between admissions and *T*_max_ graphically as a small rise and fall in admissions in cooler temperatures, and then a logarithmic trend as temperatures increased from moderate to extreme (Figure 2A). Using a nonlinear least-squares (hockey stick) estimation, we calculated a threshold temperature of 26.7°C (*p* = 0.001), above which MBD admissions increased markedly. Using iterative regressions, this threshold value remained robust to several parameter estimations.

Overall, fixed-effects Poisson regression analysis accounting for overdispersion showed a 7.3% increase in admissions during heat-wave periods compared with non–heat-wave periods in the warm season (Table 1). Results were also significant for persons ≥ 75 years of age, with an IRR of 1.183 (95% CI, 1.088–1.286), and in male admissions in the 15- to 64-year age group (Table 2).

#### Organic, including symptomatic, mental disorders (ICD-10 codes F00–F09)

In this category, encompassing dementia, cerebral disease, brain injury, and other trauma leading to cerebral dysfunction (WHO 2007), dementia (F00–F03) accounted for more than half (56.2%) of the admissions. Compared with control periods, we found that hospitalizations for organic, including symptomatic, mental disorders increased by 21.3% during heat waves (Table 1) with increases in both male and female admissions (Table 2) and in the ≥ 75-year age group. Figure 2B shows a decline in admissions associated with moderate temperatures and a linear increase as temperatures rise from moderate to high.

#### Dementia (ICD-10 codes F00–F03)

The risk of admission for dementia during heat waves increased overall by 17.4% (Table 1) and in females (Table 2). Because dementia accounted for a high proportion of organic, including symptomatic, mental disorder admissions, the shape of the relationship with *T*_max_ (Figure 2C) is almost identical to that in Figure 2B, albeit on a different scale.

#### MBDs due to psychoactive substance use (ICD-10 codes F10–F19)

In this category, the elderly (≥ 75 years of age) were the only age group to show an increase in hospitalizations during heat waves (IRR 1.567; 95% CI, 1.002–2.450), with female admissions of this age also showing a significant increase (Table 2).

#### Mood (affective) disorders (ICD-10 codes F30–F39)

Mood (affective) disorders include depression, dysthymia, mania, and bipolar affective disorders (WHO 2007). This category accounted for the largest proportion (33.9%) of MBD admissions. During heat waves, admissions increased 9.1% compared with referent periods (Table 1), with highest estimates in persons 15–64 years of age (IRR 1.102; 95% CI, 1.041–1.167).

#### Neurotic, stress-related, and somatoform disorders (ICD-10 codes F40–F48)

These disorders include anxiety disorders, panic disorder, agoraphobia, obsessive compulsive disorder, and posttraumatic stress disorder (WHO 2007). Hospitalizations increased 9.7% compared with referent periods (Table 1), and age-specific regression showed that those most at risk during heat waves were the very young and the elderly. Figure 2D shows the relationship of admissions with temperature and demonstrates a gradual increase as *T*_max_ rises above moderate.

#### Behavioral syndromes associated with physiological disturbances and physical factors (ICD-10 codes F50–F59)

This group of syndromes includes eating and sleep disorders and accounted for only 1.6% of total MBD admissions over the study period. During heat waves, admissions increased for this classification in elderly females only (Table 2).

#### Disorders of psychological development (ICD-10 codes F80–F89)

Encompassing autism and developmental disorders of speech and language, this category accounted for 0.35% of MBD admissions. We observed a 64% increase in hospitalizations overall (Table 1), whereas the 15- to 64-year age group showed a > 3-fold increase (IRR 3.187; 95% CI, 1.185–8.571) during heat waves. Figure 2E shows admissions reaching a plateau at moderate temperatures and increasing sharply in hot weather.

#### Senility (ICD-10 code R54)

Senility includes senescence, asthenia, and debility (WHO 2007), relating to mental infirmity and physical deterioration in the elderly. Although few admissions (*n* = 191) had the principal diagnosis of senility, our analysis showed a > 2-fold increase during heat waves (Table 1), with the strongest effect in the elderly (IRR 2.534; 95% CI, 1.232–5.211), and in elderly females (Table 2). Figure 2F reveals an almost U-shaped relationship, showing admissions at their lowest during mild temperatures and highest during the extremes of cold and hot weather.

### Mortalities

Mortalities attributed to the 15 categories of psychiatric disorders were relatively infrequent in Adelaide, and sample sizes during heat waves were not large. Table 3 displays regression results for the disorders showing significant increases in mortalities.

#### MBDs (ICD-10 codes F00–F99)

Over the study period, 2,599 deaths were attributed to MBDs, representing < 1/day. During heat waves, there were 70 MBD deaths, with 81% (*n* = 57) of the decedents ≥65 years of age and regression results showing a 2.4-fold increase in deaths in the 65- to 74-year age group (Table 3).

#### Organic, including symptomatic, mental disorders (ICD-10 codes F00–F09), and dementia (F00–F03)

Most MBD deaths (76%, *n* = 1,964) were due to organic, including symptomatic, mental disorders, of which dementia comprised 99% (*n* = 1,953). Of the deaths occurring during heat waves, we classified 67% (*n* = 47) as organic, including symptomatic, mental disorders, and all were due to dementia. Regression results showed that the risk of death during heat waves compared with non–heat-wave periods was significant for those 15–64 years of age and in males of this age group (Table 3).

#### Mental disorders due to psychoactive substance use (ICD-10 codes F10–F19)

During heat waves, deaths in this category increased only in females, specifically those in the 15- to 64-year age bracket (Table 3).

#### Schizophrenia and schizotypal and delusional disorders (ICD-10 codes F20–F29)

Analysis of nonstratified data showed deaths due to schizophrenia and schizotypal and delusional disorders increased > 2-fold during heat-wave periods (Table 3). This was more pronounced in those ≥ 75 years of age and in males of this age group. Exploratory graphical analysis showed mortality to be lowest at moderate temperatures and highest when temperatures exceeded 35°C (data not shown), although low counts may have compromised the integrity of the association.

## Discussion

This study builds on our previous investigation into the acute effects of heat waves, where among the range of diagnoses examined, mental disorders was the only group in which we observed increases in both hospital admissions and (age-specific) mortality in this temperate city (Nitschke et al. 2007). Other published literature has also identified persons with mental illness as being a heat-susceptible subgroup of the population (Basu and Samet 2002; Kaiser et al. 2001; Kovats and Ebi 2006; Naughton et al. 2002). However, to our knowledge, this study is the first endeavor to comprehensively characterize specific disorders that contribute to increased psychiatric morbidity and mortality during heat waves. Our results suggest that several mental illnesses may be sensitive to exposure to high ambient temperatures. We have shown that hospital admissions for MBDs increased at temperatures above 26.7°C, and they also increased by 7.3% during heat waves compared with control periods. Specific nosologic subgroups for which an increase in admissions was evident include organic, including symptomatic, mental disorders; dementia; mood (affective) disorders; neurotic, stress-related, and somatoform disorders; and disorders of psychological development. Although classified separately to MBDs, senility was included in this study because a cognitive impairment of the elderly and admissions for senility increased > 2-fold during heat-wave periods. Hospitalizations for behavioral syndromes associated with physiological disturbances and physical factors, and disorders due to psychoactive substance use, increased for specific age and sex categories.

Evidence has shown that having a pre-existing psychiatric illness can more than triple the risk of death during a heat wave (Bouchama et al. 2007). In the present study, we observed an increase in deaths classified as MBDs in the 65- to 74-year age group during heat waves. Additionally, deaths due to schizophrenia and schizotypal and delusional disorders increased > 2-fold, and those due to psychoactive substance use increased in all females and in females 15–64 years of age. Dementia deaths increased in the 15- to 64-year age group only, indicating the susceptibility of persons with early-onset dementia. Because our mortality data was limited by small sample sizes in most categories, the association between heat waves and mental health mortalities warrants cautious interpretation of some results, particularly where stratification of data compromised statistical power.

Two issues concern the effects of heat on psychiatric illness: first, the nature of the psychiatric condition can be a risk factor for heat-related morbidity and mortality, and second, heat can be a risk factor for the exacerbation of the condition. Because heat-related illness is often underdiagnosed (Varghese et al. 2005), individuals in our study with a case history of mental illness may have been suffering the effects of heat rather than (or as well as) the effects of their disorder, the latter taking likely precedence for classification purposes.

That mental illness can be a risk factor for heat-related morbidity and mortality is plausible for physiological and behavioral reasons (Faunt et al. 1995). Many medications used in psychiatry increase vulnerability to heat-related morbidity by altering the body’s ability to thermoregulate (Batscha 1997; Conti et al. 2007; Flynn et al. 2005) because of pharmacologic effects on the parasympathetic pathway (Martin-Latry et al. 2007). Drugs such as anti-psychotics, anticholinergics, antidepressants, sedatives, and mood stabilizers that impair sweating and/or increase heat production (Batscha 1997) are used in the treatment of such conditions as dementia, Alzheimer’s disease, psychosis, mood disorders, personality disorders, and anxiety disorders. The inherent nature of these illnesses may also contribute to vulnerability during hot weather. Cognitive awareness of environmental conditions and the ability to undertake adaptive behaviors such as increased fluid intake or wearing appropriate clothing (Bark 1998; Martin-Latry et al. 2007) are important coping mechanisms that may be compromised in those with disabling mental illnesses such as Alzheimer’s disease, dementia, senility, psychosis (Martin-Latry et al. 2007), schizophrenia (Bark 1998), and developmental disabilities. Additionally, severe or core activity limitations often presenting in the mentally ill (Australian Bureau of Statistics 2006) can determine their degree of dependence and be a contributing factor to heat susceptibility (Davido et al. 2006). This notion is supported by our findings of increased admissions for individuals with senility, confirming the susceptibly of the frail elderly to heat-related illnesses or death (Conti et al. 2007; Davido et al. 2006; Faunt et al. 1995; Kovats and Ebi 2006; Semenza et al. 1996).

In contrast to the findings of others who reported a correlation of schizophrenia admissions with environmental temperature (Shiloh et al. 2005), we found no increase in admissions during heat waves for the nonstratified or age- and sex-stratified data. However, we observed a significant increase in mortalities attributed to schizophrenia and schizotypal and delusional disorders. A coroner’s study of 18 heat-related deaths during the 1999 Cincinnati heat wave also found schizophrenia to be a risk factor for mortality, with eight decedents having a mental illness, four of whom had schizophrenia (Kaiser et al. 2001). Assumption as to the nature of our varying morbidity and mortality rates in this category remains highly speculative. First, antipsychotic medications that impair thermoregulation may increase the risk of death in psychiatric patients during heat waves (Bark 1998), although studies have shown patients were at risk in the 1950s before the introduction of antipsychotic drugs, suggesting that both schizophrenia and its medication increase the risk of heat-related death (Bark 1998). Second, it is possible that, for these individuals, death may have occurred either rapidly before admission to hospital (Kovats et al. 2004) or under conditions of social isolation (Naughton et al. 2002; Semenza et al. 1996). Furthermore, consequential factors of living with such a debilitating mental illness include socioeconomic deprivation, solitary lifestyle, poor general health, and chronic illness, all of which are risk factors for heat-related mortality (Basu and Samet 2002; Semenza et al. 1996).

In addition to schizophrenia, the ICD-10 category F20–F29 includes delusional disorders (codes F22.0–F22.9), a subcategory not investigated independently here. Altered mental status can be a symptom of heatstroke, heat stress, or hyperthermia (Bouchama and Knochel 2002; Davido et al. 2006; Dematte et al. 1998), often presenting as delirium or disorientation (Bouchama and Knochel 2002; Dematte et al. 1998). Heatstroke is common in the elderly, has a high fatality rate because of multiorgan dysfunction (Varghese et al. 2005), and often goes either undiagnosed (Bark 1998; Bouchama and Knochel 2002; Varghese et al. 2005) or not recorded as the underlying cause of death (Basu and Samet 2002; Hajat et al. 2007). Our results showed that eight of the nine patients whose deaths during heat waves were attributed to schizophrenia and schizotypal and delusional disorders were ≥ 75 years of age. That our results indicated more than twice the relative risk of death for these elderly persons during heat waves compared with control periods raises the possibility that they may have had delusional disorders rather than schizophrenia reported as the underlying cause of death. However, to avoid speculation, further investigation would be required at an individual rather than population level of data.

A considerable body of literature has shown that dementia and cognitive impairment confer vulnerability to extreme heat (Conti et al. 2007; Faunt et al. 1995; Green et al. 2001; Kaiser et al. 2001). Psychotropic drugs with thermoregulatory side effects (Bark 1998; Conti et al. 2007; Kaiser et al. 2001; Martin-Latry et al. 2007) are commonly administered to dementia patients (Kim and Whall 2006) and may additionally modify vigilance levels (Martin-Latry et al. 2007) and cognitive awareness of the need for adaptive behaviors to avoid thermal stress. Use of these medications has been shown to be associated with a significantly increased risk of heat-related hospitalization (Martin-Latry et al. 2007) and death during heat waves (Bouchama et al. 2007). Our results showed an overall increase in hospital admissions for dementia during heat waves and an increase in dementia deaths in the 15- to 64-year age group. Although this latter finding was surprising, it is possible that older persons with dementia are more likely to reside in elderly care facilities, thereby lowering their risk of death during heat waves (Bouchama et al. 2007). With an estimated 231% rise in new dementia cases per year by 2050 (Jorm et al. 2005), these findings indicating that dementia may be a heat-sensitive illness suggest that the future burden on the health sector will be substantial.

Evidence has shown that elevated temperatures may exacerbate psychiatric conditions. Environmental changes can affect mental health (Woodruff et al. 2006), with excessive heat and humidity reported to be major influences affecting mood and behavior (Morrissey et al. 1996). Fluctuations in weather have been noted to cause an increase in the incidence of mental stress, depression (McMichael et al. 1996), and suicide (Page et al. 2007). As temperatures rise to extreme, stresses of everyday home, social, or work life are likely to be compounded by lethargy, lack of sleep, and the inability to function normally during oppressively hot conditions. For those predisposed to acute or chronic mental problems, failure to gain relief from the heat for extended periods of time may trigger irritability and episodic psychological distress, accompanied by risk behaviors such as excess alcohol consumption, violence, and aggression (Page et al. 2007). With heat waves predicted to occur more frequently [Intergovernmental Panel on Climate Change (IPCC) 2007; McMichael and Beers 1994; McMichael et al. 1996], an increase in mental problems may be an indirect public health consequence of a warming climate (McMichael et al. 1996). The effects of sustained heat and humidity, accompanied by drought, water restrictions, bushfires (Selvey and Sheridan 2002), and power outages (Essential Services Commission of South Australia 2006), are likely to have marked effects on the mental health of both rural (Morrissey and Reser 2007; Research Australia 2007) and urban communities, with possible increases in the incidence of episodic or chronic stress, despair and depression, and health-damaging personal behaviors (Research Australia 2007). This could have significance across the public health sector and implications for law enforcement and social cohesion.

Statistics indicate an increase in reported mental illness in Australia, with 11% of persons reporting a long-term mental or behavioral problem in 2004–2005, up from 5.9% in 1995 (Australian Bureau of Statistics 2006). Furthermore, as a consequence of the demographic shift, age-related mental impairments will be more common in years to come, placing financial strains on the economy. Recent figures show that expenditures on mental health services (excluding spending on Alzheimer’s and other nervous system disorders) totaled AU$3.0 billion in 2000–2001 (Australian Institute of Health and Welfare 2004). To mitigate the effect of heat on the vulnerable, including the mentally ill, relevant health promotion and health intervention strategies should be considered, including the formulation of heat-wave response plans incorporating heat alerts to the public and health authorities when excessively hot conditions are expected (Research Australia 2007). Similar heat-health action plans (WHO 2008) are now in place in most European cities after the health tragedy of the 2003 heat wave (Linares and Diaz 2008).

Although population acclimatization to a warmer climate will undoubtedly occur and consequently lessen the impact of heat waves, adaptation strategies may, for behavioral and physiologic reasons, be less achievable in persons with mental illnesses. Our study design prevented us from examining possible adaptation over the study period or any potential differences in health impacts between early-and late-season heat waves. The assumption of nonvarying population response to heat over the study period may be one of several limitations of the study.

Furthermore, our data assume correct ICD coding for MBDs. Errors in diagnostic classification would influence accuracy of effect estimates, particularly in cases of acute rather than chronic psychiatric illness. Substantial changes that occurred in the classification of diagnostic criteria for mental disorders in the transition from ICD-9 to ICD-10 may have increased the risk of misclassification by coders. For example, in the ICD-10, the classification of “dementia in Alzheimer’s disease” (F00–F00.9) is not listed in the same chapter as “Alzheimer’s disease” (G30–G30.9), highlighting the ease with which inaccuracies in coding can occur.

Second, we acknowledge the small sample size in some categories, which limits statistical power. Further, inflation of type 1 error may arise when multiple comparisons are undertaken as in our study. Accordingly, we advise cautious interpretation of some results, especially mortality results.

Finally, in an ecological study such as ours, it is not possible to determine whether those with a mental or behavioral disorder were admitted or died because of the effects of heat, or an increase in severity of their mental condition. Moreover, persons with psychiatric illnesses are more likely to have preexisting comorbidities and live in financial hardship and social isolation (Martin-Latry et al. 2007), risk factors we could not account for in this study. Further research using clinical records would reveal medication history and prognostic outcomes, thereby adding additional insights into the susceptibility of the mentally ill to the effects of heat.

The present study has shown that heat waves pose a salient risk to the well-being of those with mental and cognitive disorders. The extent of the future risk will be largely determined by planning and mitigation. With heat extremes very likely to occur more frequently (IPCC 2007) in countries around the globe, our findings have relevance on a local and international scale. Improvements in the management and care of the mentally ill will need to be addressed to avoid major economic and social costs to society of heat-related psychiatric morbidity and mortality exacerbated by climate change.

## Figures and Tables

**Table 1:** Cause-specific MBD hospital admissions associated with heat waves in Adelaide, 1993–2006.

ICD-10 code	Details	IRR	95% CI
F00–F99	MBDs	1.073	1.017–1.132
F00–F09	Organic, including symptomatic, mental disorders	1.213	1.091–1.349
F00–F03	Dementia	1.174	1.017–1.355
F10–F19	Mental and behavioral disorders due to psychoactive substance use	1.005	0.913–1.105
F20–F29	Schizophrenia, schizotypal, and delusional disorders	1.034	0.969–1.102
F30–F39	Mood (affective) disorders	1.091	1.004–1.185
F40–F48	Neurotic, stress-related, and somatoform disorders	1.097	1.018–1.181
F50–F59	Behavioral syndromes associated with physiological disturbances and physical factors	0.875	0.678–1.130
F60–F69	Disorders of adult personality and behavior	1.049	0.905–1.214
F70–F79	Mental retardation	0.737	0.268–2.026
F80–F89	Disorders of psychological development	1.641	1.086–2.480
F90–F98	Behavioral and emotional disorders with onset usually occurring in childhood and adolescence	0.578	0.349–0.955
G30–G30.9	Alzheimer’s disease	1.154	0.894–1.489
G31.1	Senile degeneration of brain, not elsewhere classified	7.727	0.701–85.217
R54	Senility	2.366	1.200–4.667

**Table 2:** Cause-specific hospital admissions stratified by age and sex.

		All ages		15–64 years		65–74 years		≥75 years	
Diagnosis	Sex	IRR	95% CI	IRR	95% CI	IRR	95% CI	IRR	95% CI
MBDs	M	1.077	1.030–1.126	1.059	1.008–1.112	1.139	0.950–1.365	1.171	1.017–1.348
	F	1.070	0.997–1.148	1.043	0.966–1.127	1.092	0.942–1.265	1.190	1.073–1.320
Organic, including symptomatic, mental disorders	M	1.177	1.000–1.385	1.139	0.772–1.681	1.447	1.023–2.047	1.107	0.897–1.366
	F	1.242	1.079–1.429	1.300	0.871–1.941	1.065	0.701–1.618	1.262	1.075–1.483
Dementia	M	1.118	0.894–1.399	1.282	0.549–2.991	1.162	0.724–1.864	1.091	0.836–1.424
	F	1.215	1.008–1.466	1.396	0.596–3.268	1.249	0.745–2.095	1.201	0.976–1.477
Disorders due to psychoactive substance use	M	1.026	0.913–1.153	1.022	0.904–1.155	1.040	0.603–1.795	0.978	0.473–2.022
	F	0.965	0.819–1.137	0.916	0.766–1.094	1.100	0.530–2.281	2.359	1.332–4.180
Schizophrenia, schizotypal, and delusional disorders	M	1.048	0.966–1.136	1.041	0.958–1.131	1.018	0.585–1.770	1.533	0.761–3.085
	F	1.010	0.880–1.160	0.985	0.842–1.152	1.278	0.900–1.816	1.111	0.684–1.804
Mood (affective) disorders	M	1.085	0.995–1.182	1.057	0.960–1.164	1.093	0.809–1.476	1.271	0.980–1.649
	F	1.093	0.973–1.229	1.118	1.001–1.249	1.092	0.819–1.457	0.928	0.763–1.130
Neurotic, stress-related, and somatoform disorders	M	1.151	1.031–1.285	1.118	0.993–1.258	0.982	0.542–1.777	1.520	0.937–2.465
	F	1.054	0.953–1.166	1.037	0.927–1.160	1.059	0.708–1.585	1.276	0.926–1.759
Syndromes associated with physiological development, disturbances, and physical factors	M	0.777	0.315–1.917	0.840	0.305–2.309	—		—	
	F	0.885	0.678–1.155	0.825	0.620–1.099	—		5.894	1.067–32.565
Disorders of psychological development	M	1.480	0.877–2.498	3.383	1.247–9.180	—		—	
	F	1.985	1.013–3.891	—		—			
Behavioral and emotional disorders, childhood and adolescence onset	M	0.448	0.221–0.908	0.457	0.144–1.453	3.691	0.421–32.327	—	
	F	0.814	0.396–1.670	0.504	0.122–2.092	—		—	
Alzheimer’s disease	M	1.009	0.664–1.535	—		1.120	0.482–2.606	1.006	0.620–1.633
	F	1.258	0.912–1.735	1.133	0.263–4.872	0.629	0.195–2.027	1.377	0.976–1.944
Senility	M	0.594	0.080–4.404	—		0.770	0.103–5.779		
	F	3.590	1.696–7.600	—		3.488	0.387–31.422	3.605	1.623–8.004

Abbreviations: F, female; M, male.

**Table 3:** Details of mental and behavioral mortalities associated with heat waves.

Diagnosis	Group	IRR	95% CI	*p*-Value
All MBDs	65–74 years	2.395	1.165–4.922	0.017
Dementia	15–64 years	5.058	1.205–21.232	0.027
	Males 15–64 years	12.731	2.064–78.516	0.006
Disorders due to psychoactive substance use	Females, all ages	3.098	1.342–7.155	0.008
	Females 15–64 years	3.211	1.297–7.948	0.012
Schizophrenia, schizotypal, and delusional disorders	All ages	2.079	1.045–4.138	0.037
	≥75 years	2.111	1.018–4.380	0.045
	Males, all ages	4.051	1.386–11.840	0.011
	Males ≥75 years	5.255	1.752–15.758	0.003
